# Effect of *Cudrania tricuspidata* and Kaempferol in Endoplasmic Reticulum Stress-Induced Inflammation and Hepatic Insulin Resistance in HepG2 Cells

**DOI:** 10.3390/nu8010060

**Published:** 2016-01-21

**Authors:** Ok-Kyung Kim, Woojin Jun, Jeongmin Lee

**Affiliations:** 1 Department of Medical Nutrition, Kyung Hee University, Yongin 17104, Korea; eorkfgod@naver.com; 2 Division of Food and Nutritional Science, Chonnam National University, Gwangju 61186, Korea; wjjun@chonnam.ac.kr

**Keywords:** *Cudrania tricuspidata*, obesity, insulin resistance, ER stress, inflammation

## Abstract

In this study, we quantitated kaempferol in water extract from *Cudrania tricuspidata* leaves (CTL) and investigated its effects on endoplasmic reticulum (ER) stress-induced inflammation and insulin resistance in HepG2 cells. The concentration of kaempferol in the CTL was 5.07 ± 0.08 mg/g. The HepG2 cells were treated with 300 µg/mL of CTL, 500 µg/mL of CTL, 1.5 µg/mL of kaempferol or 2.5 µg/mL of kaempferol, followed immediately by stimulation with 100 nM of thapsigargin for ER stress induction for 24 h. There was a marked increase in the activation of the ER stress and inflammation response in the thapsigargin-stimulated control group. The CTL treatment interrupted the ER stress response and ER stress-induced inflammation. Kaempferol partially inhibited the ER stress response and inflammation. There was a significant increase in serine phosphorylation of insulin receptor substrate (IRS)-1 and the expression of C/EBPα and gluconeogenic genes in the thapsigargin-stimulated control group compared to the normal control. Both CTL and kaempferol suppressed serine phosphorylation of IRS-1, and the treatments did not interrupt the C/EBPα/gluconeogenic gene pathway. These results suggest that kaempferol might be the active compound of CTL and that it might protect against ER stress-induced inflammation and hyperglycemia.

## 1. Introduction

The endoplasmic reticulum (ER) is one of the largest cytoplasmic organelles in eukaryotic cells. It has important functions in calcium storage, lipid synthesis and protein folding. All proteins enter the secretory pathway through the ER, where they are then folded and modified by asparagine-linked glycosylation and disulfide bonds [1]. A physiological imbalance between the cellular demand for protein folding and the capacity of the ER to promote protein maturation results in an accumulation of unfolded proteins in the ER lumen [2,3]. The accumulation of unfolded proteins triggers an unfolded protein response (UPR). The UPR is initiated by three ER transmembrane proteins: protein kinase RNA-like endoplasmic reticulum kinase (PERK), activating transcription factor 6 (ATF6) and inositol-requiring enzyme 1 (IRE1). Three main ER transmembrane protein-signaling systems induce the degradation of the unfolded proteins and the expression of chaperones, such as 78-κDa glucose-regulated protein (GRP-78), which assist in protein folding. When the UPR is unable to prevent the accumulation of unfolded proteins, it can result in the ER stress response [2,3].

Recent studies have reported that ER stress can lead to inflammation and insulin resistance [4,5,6,7]. Studies have reported multiple apoptotic pathways, including activation of the ccaat-enhancer-binding proteins (C/EBP) homologous protein (CHOP), mediated by PERK and ATF6-N [8,9]. ER stress-induced CHOP expression plays a role in cytokine-induced pro-inflammatory responses and the pathogenesis of inflammation [10]. In addition, ER stress-induced IRE1 can be required for the activation of the nuclear factor kappaB (NF-κB) in response to ER stress [7]. The activation of IRE1 also leads to the phosphorylation and activation of the c-Jun N-terminal kinase (JNK) [9]. The activation of the JNK pathway is associated with the development of insulin resistance via serine phosphorylation of insulin receptor substrate (IRS)-1, which leads to the suppression of insulin receptor signaling [11,12]. ER stress-induced inflammation and insulin resistance play a central role in the pathogenesis of metabolic disorders, and ER stress can be a major factor in the mechanism of obesity-induced insulin resistance [13,14,15]. Therefore, it is important to study the natural plan or phytochemicals that inhibit the ER stress and can prevent the development of risk factors for many diseases.

Recent studies have revealed that *Cudrania tricuspidata* (CT) has antioxidant, anti-obesity, anti-inflammation and anti-hyperglycemic effects [16,17,18,19,20]. CT, of the family Moraceae, is mainly found in East Asia. In China, an herbal medicine dictionary has that CT treats furuncle and eczema and also accelerate blood circulation, pain relief and the treatment of mumps, tuberculosis and contusions [16,17,18]. In a comparison of the total polyphenol content and antioxidant effects of parts of the leaves, stem and fruit of CT, the polyphenol levels and antioxidant effects were reported to be the highest in the leaves [21]. Studies have shown that phenolic compounds, including various types of xanthones, as well as flavonoids, such as kaempferol, were the major constituents of CT leaves and that the constituents of CT leaves had antioxidant effects [16,22]. Kaempferol, kaempferol 7-*O*-glucopyranoside, naringin 7-*O*-glucopyranoside, β-sitosterol, β-sitosterol glucoside and 5-*O*-methyl genistein also have been isolated from the stems and leaves of CT [16,22]. In this study, we quantitated kaempferol in water extracted from CT leaves (CTL) and investigated the effects of kaempferol on inflammation and hepatic insulin resistance in ER stress-induced HepG2 cells.

## 2. Experimental Section

### 2.1. CTL Extract Preparation

CT was obtained from Goseong Agricultural Corporation and extracted. The dried leaves (50 g) were boiled with distilled water (1 L) at 100 °C for 4 h in a reflux apparatus and filtered. The extracts were concentrated *in vacuo* and lyophilized. The dried CTL were obtained at a yield of 15.6% and kept in an air-tight and light-protected container at −20 °C until used.

### 2.2. Quantitative Analysis of Kaempferol

Quantitative determination of kaempferol in the CTL water extract was performed using high-performance liquid chromatography (HPLC) (HPLC system, Agilent 1260 Infinity, Santa Clara, CA, USA). A reference standard stock solution containing kaempferol (Sigma, St. Louis, MO, USA) was used. The CTL (30 mg) was diluted with 10 mL solution (EtOH:DW:HCl = 50:20:8). The solution was refluxed for 1 h at 100 °C for hydrolysis. The solution was extracted with methanol and filtered with a 0.45 μm syringe filter before injection into the HPLC column. A Capcellpak C18 MG120 column (4.6 × 250 mm, 5 μm) was used for the separation. The mobile phase consisted of methanol and 0.85% phosphoric acid (1:1). The injection volume was 5 µL, and the flow rate was 1.0 mL/min. The UV detection wavelength was set at 370 nm, and the column temperature was 35 °C. The amount of kaempferol in the CTL water extract was determined from a calibration curve obtained by the concentration of kaempferol standard against the peak area.

### 2.3. Cell Culture and Cell Viability

HepG2 cells were obtained from the American type culture collection (ATCC, Manassas, VA, USA). The cells were maintained in Dulbecco’s minimal essential medium (DMEM, Hyclone Laboratories, Logan, UT, USA) containing 10% fetal bovine serum (FBS, Hyclone Laboratories, Logan, UT, USA), 100 mg/L of penicillin-streptomycin and 2 mmol/L of glutamine (Hyclone Laboratories, Logan, UT, USA). The cells were maintained at 37 °C under a humidified atmosphere of 5% CO_2_. The medium was refreshed approximately three times a week.

The cell viability was assessed using 3-(4,5-dimethylthiazol-2yl-)-2,5-diphenyl tetrazolium bromide (MTT) (Sigma Aldrich, St. Louis, MO, USA).

The HepG2 cells were cultured at a concentration of 3 × 10^4^ cells/well in a 96-well tissue culture plate with CTL at various concentrations of 100–800 µg/mL. After incubation for 24 h, 20 μL of the MTT solution (5 mg/mL in PBS as the stock solution) were added into each well, and the cells were incubated again at 37 °C for 3 h. The supernatants were removed, and DMSO (200 μL) was then added to each well. The plates were read at 560 nm to obtain the percentage of viable cells.

### 2.4. Cell Treatments

The HepG2 cells were seeded at a concentration of 3 × 10^5^ cells/well in 6-well tissue culture dishes and incubated to proliferate for 24 h. The cells were then treated with CTL 300 µg/mL (CTL300), CTL 500 µg/mL (CTL500), kaempferol 1.5 µg/mL (K1.5) or kaempferol 2.5 µg/mL (K2.5), immediately followed by stimulation with 100 nM thapsigargin for ER stress induction for 24 h.

### 2.5. Protein Extraction and Western Blot Analysis

The cells were harvested, lysed in a CelLytic™ MT cell lysis reagent (Sigma Aldrich, St. Louis, MO, USA) and centrifuged at 12,000× *g* for 20 min at 4 °C. Protein samples (100 μg protein/lane) were dissolved in NuPAGE^®^ LDS sample buffer 4× (Life Technologies, Gaithersburg, MD, USA), separated on SDS-polyacrylamide gel and transferred to nitrocellulose membranes (Bio-Rad Laboratories, Hercules, CA, USA). The membranes were incubated in a blocking solution and then incubated for 12 h at 4 °C with anti-total-eIF2α (1:500), anti-phospho-eIF2α (1:500), anti-total-IRE1α (1:1000, Novus Bio, Littleton, CO, USA), anti-phospho-IRE1α, anti-total JNK (1:1000), anti-phospho-JNK (1:1000), anti-total NF-κB (1:1000) anti-phospho-NF-κB (1:1000), anti-total IRS-1 (1:1000) and anti-phospho-IRS-1 (serine, 1:1000) antibodies. All of the antibodies were purchased from Cell Signaling (Beverly, MA, USA), except for the anti-total-IRE1α antibody (Novus Biologicals, Littleton, CO, USA). After incubation, the membranes were incubated with a secondary antibody (anti-rabbit IgG HRP-linked antibody, 1:5000, Cell Signaling, Beverly, MA, USA) for 1 h at room temperature. Protein bands were developed using Super Signal West Dura Extended Duration Substrate (Pierce, Milwaukee, WI, USA) and visualized with the ChemiDoc imaging system from Bio-Rad Laboratories (Hercules, CA, USA).

### 2.6. Isolation of Total RNA and Real-Time PCR

The cells were lysed in the presence of a lysis buffer (RLT, Qiagen, Valencia, CA, USA), including 1% β-mercaptoethanol. Total RNA was extracted using the RNeasy Mini kit (Qiagen, Valencia, CA, USA), and complementary DNA was synthesized from 400 ng of purified total RNA in 20 μL of reaction buffer using the iScript™ cDNA Synthesis Kit (Bio-Rad Laboratories, Hercules, CA, USA). Real-time PCR (Applied Biosystems, Foster City, CA, USA) was performed using 10 ng cDNA with the SYBR Green PCR Master Mix (iQ SYBR Green Supermix, Bio-Rad Laboratories, Hercules, CA, USA). The cDNA was amplified for 45 cycles of denaturation (95 °C for 30 s), annealing (58 °C for 30 s) and extension (72 °C for 45 s) with specific primers (Table 1). The results of the real-time RT-PCR were processed with the 7500 System SDS software Version 1.3.1 (Applied Biosystems, Foster City, CA, USA). The same software was used to process the quantitative data.

**Table 1:** Primer sets used for real-time PCR.

Gene	Accession Number	Sequence	
GAPDH (H)	M33197	F	5′-ATG GAA ATC CCA TCA CCA TCT T-3′
		R	5′-CGC CCC ACT TGA TTT TGG-3′
ATF4 (H)	BC016855	F	5′-GAG GTG GCC AAG CAC TTC AA-3′
		R	5′-GCC CGC CTT AGC CTT GTC-3′
CHOP (H)	S40706	F	5′-CTC TGA TTG ACC GAA TGG TGA A-3′
		R	5′-GGG ACT GAT GCT CCC AAT TG-3′
XBP-1 (H)	NM005080	F	5′-CCT GAG CCC CGA GGA GAA-3′
		R	5′-GGC AGT CTG AGC TGC TAC TCT GT-3′
GRP78 (H)	X87949	F	5′-TGG CGG AAC CTT CGA TGT-3′
		R	5′-GCC ACA ACT TCG AAG ACA CCA T-3′
TNF-α (H)	NM000594	F	5′-CCA CTT CGA AAC CTG GGA TTC-3′
		R	5′-TTA GTG GTT GCC AGC ACT TCA-3′
IL-1β (H)	NM000576	F	5′-TTA AAG CCC GCC TGA CAG A-3′
		R	5′-GCG AAT GAC AGA GGG TTT CTT AG-3′
IL-6 (H)	M54894	F	5′-AGG GCT CTT CGG CAA ATG TA-3′
		R	5′-GAA GGA ATG CCC ATT AAC AAC AA-3′
C/EBPα (H)	NM005194	F	5′-AAC TTG TGC CTT GGA AAT GCA -3′
		R	5′-CAC GAT TTG CTC CCC CTA CTC-3′
PEPCK (H)	NM002591	F	5′-TGG GCT CGC CTC TGT CA-3′
		R	5′-CCA CCA CGT AGG GTG AAT CC-3′
G6Pase (H)	U01120	F	5′-GAG TGG AGT GGC ACG ATC TTG-3′
		R	5′-GAC ATG AGA ATC GCT TGA ACC A-3′

F: Forward; R: Reverse.

### 2.7. Statistical Analysis

All of the data were expressed as the mean ± standard deviation (SD). The significance of the treatment effects was analyzed using Duncan’s multiple-range tests after a one-way ANOVA using SPSS statistical procedures for Windows (SPSS PASW Statistic 20.0, SPSS Inc. Chicago, IL, USA). Statistical significance was considered at the *p* < 0.05 level.

## 3. Results

### 3.1. Determination of the Kaempferol Content

**Figure 1:**
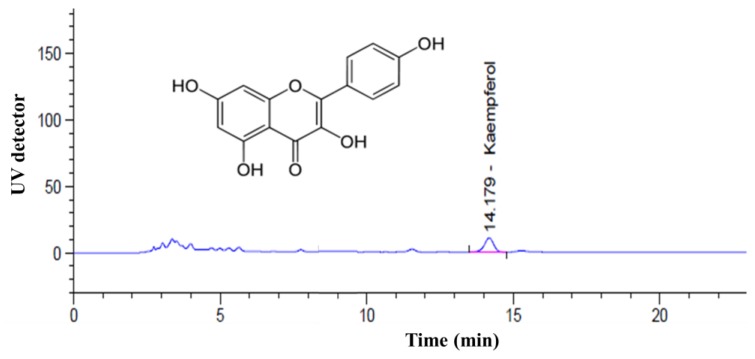
High-performance liquid chromatography (HPLC) analysis of kaempferol in *Cudrania tricuspidata* leaves (CTL).

Figure 1 shows the results of the HPLC analysis of the kaempferol content in water extracted from CTL. The concentration of kaempferol in the water extracted from CTL was calculated using standard curves. The concentration of kaempferol was 5.07 ± 0.08 mg/g. According to this result, we then investigated the effects of different levels of kaempferol (1.5 μg/mL and 2.5 μg/mL) and different concentrations of CTL (300 μg/mL and 500 μg/mL) on ER stress-induced HepG2 cells.

### 3.2. Determination of the Kaempferol Content

**Figure 2:**
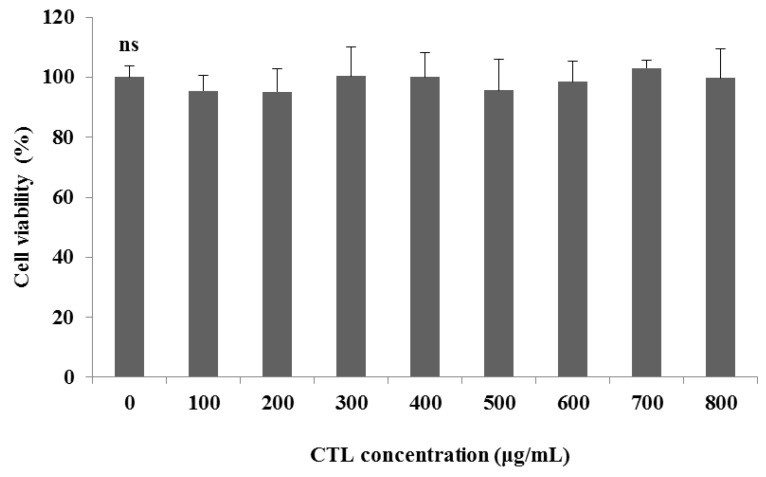
Viability of HepG2 cells following 24 h of treatment with different concentrations of CTL. The data are expressed as the mean ± SD (*n* = 3), and significant differences were analyzed using Duncan’s multiple-range test. ns = not significant.

CTL showed no signs of cytotoxicity at the tested concentrations (100–800 µg /mL) in the HepG2 cells (Figure 2). We then investigated the effect of 300 μg/mL and 500 μg/mL of CTL on the thapsigargin-stimulated HepG2 cells.

### 3.3. Effect of CTL and Kaempferol on ER Stress in ER Stress-Induced HepG2 Cells

**Figure 3:**
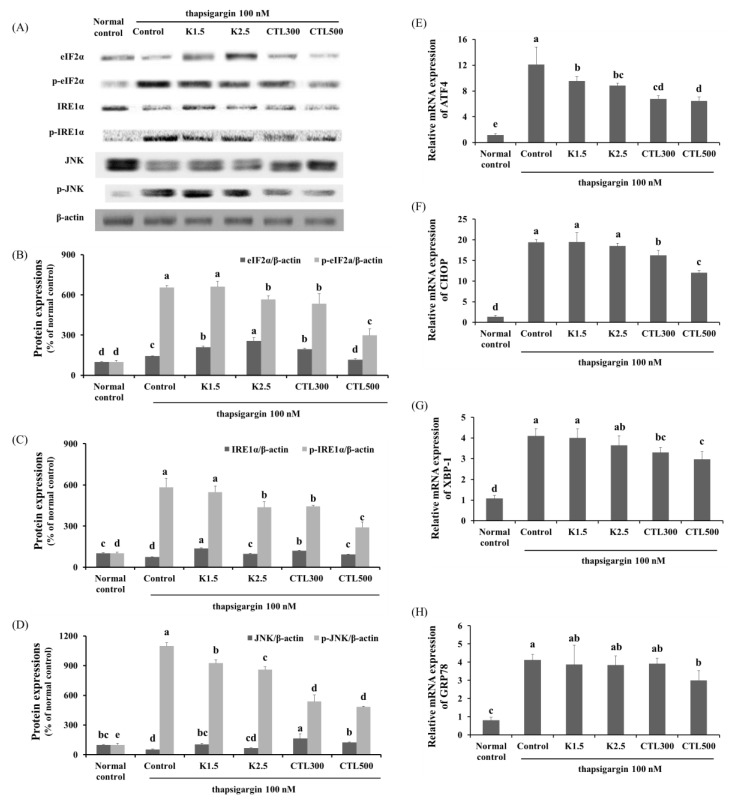
Effect of CTL and kaempferol (K) on ER stress in thapsigargin-induced HepG2 cells. (**A**) Representative Western blots for total protein and phosphorylation of eIF2α, IRE-1α and JNK in thapsigargin-induced HepG2 cells with CT water extract or kaempferol; (**B**) densitometric analysis of the phosphorylation of eIF2α, (**C**) IRE-1α and (**D**) JNK; mRNA expression of (**E**) ATF4, (**F**) CHOP, (**G**) XBP-1 and (**H**) GRP78 in thapsigargin-induced HepG2 cells with CT water extract or kaempferol. The data are expressed as the mean ± SD (*n* = 3). The different letters show the significant difference at *p* < 0.05 as determined by Duncan’s multiple-range test.

The expression of phosphorylated eIF2α, IRE1α and JNK was determined by Western blot analysis, and mRNA expression of ATF4, CHOP, XBP-1 and GRP78 was determined by real-time PCR in thapsigargin-induced HepG2 cells with CT water extract or kaempferol. When compared to a normal control group, the expression of eIF2α, IRE1α and JNK phosphorylation was increased significantly in the thapsigargin-stimulated HepG2 cells. In addition, the expression of ATF4, CHOP, X-box binding protein 1 (XBP-1) and GRP78 was significantly increased in the thapsigargin-stimulated control group compared to the normal control group. When the thapsigargin-stimulated HepG2 cells were treated with kaempferol 2.5 μg/mL, the phosphorylation of eIF2α, IRE1α and JNK, as well as the mRNA expression of ATF4 decreased significantly. The treatments with CTL300 showed significant decreases in the phosphorylation of eIF2α, IRE1α and JNK and significant decreases in the mRNA expression of ATF4, CHOP and XBP-1 compared to the thapsigargin-stimulated control group. The treatments with CTL500 caused significant decreases in the phosphorylation of eIF2α, IRE1α and JNK and significant decreases in the mRNA expression of ATF4, CHOP, XBP-1 and GRP78 compared to the thapsigargin-stimulated control group (Figure 3) (*p* < 0.05).

### 3.4. Effect of CTL and Kaempferol on Inflammation in the ER Stress-Induced HepG2 Cells

**Figure 4:**
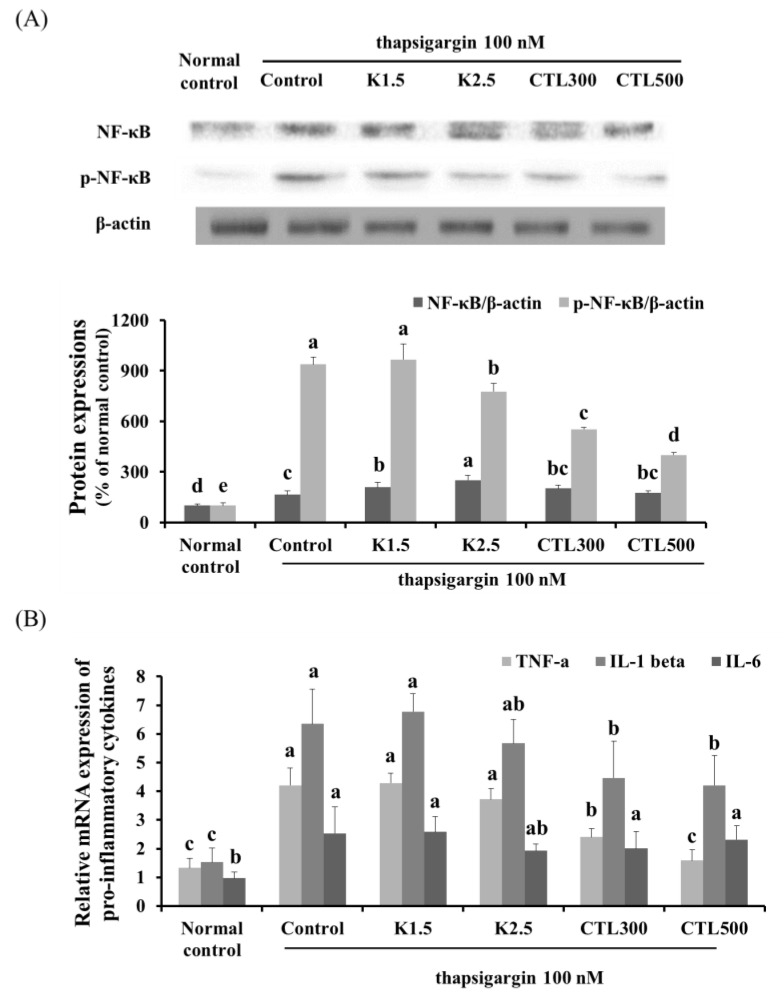
Effect of CTL and kaempferol on inflammation in thapsigargin-induced HepG2 cells. (**A**) Representative Western blots for total protein and phosphorylation of NF-κB in thapsigargin-induced HepG2 cells with CT water extract or kaempferol; (**B**) mRNA expression of pro-inflammatory cytokines (TNF-α, IL-1β and IL-6) in thapsigargin-induced HepG2 cells with CT water extract or kaempferol. The data are expressed as the mean ± SD (*n* = 3). The different letters show a significant difference at *p* < 0.05 as determined by Duncan’s multiple-range test.

The expression of phosphorylated NF-κB was determined by Western blot analysis, and mRNA expression of pro-inflammatory cytokines was determined by real-time PCR in thapsigargin-induced HepG2 cells with CT water extract or kaempferol. The thapsigargin-stimulated HepG2 cells showed a marked increase in the expression of NF-κB phosphorylation compared to the normal HepG2 cells. In addition, significant increases in the mRNA expression of pro-inflammatory cytokines (tumor necrosis factor (TNF)-α, interleukin (IL)-1β and IL-6) were observed in the thapsigargin-stimulated control group compared to the normal control group. NF-κB phosphorylation decreased significantly in the thapsigargin-stimulated HepG2 cells treated with kaempferol 2.5 μg/mL compared to the thapsigargin-stimulated control group. However, there was no significant difference in the expression of pro-inflammatory cytokines in the thapsigargin-stimulated HepG2 cells treated with kaempferol compared to the thapsigargin-stimulated control group. The thapsigargin-stimulated HepG2 cells treated with CTL showed a significant decrease in the expression of NF-κB phosphorylation in a dose-dependent manner. Moreover, the thapsigargin-stimulated HepG2 cells treated with CTL showed a significant decrease in TNF-α and IL-1β, but no significant difference in the expression of IL-6 compared to the thapsigargin-stimulated control group (Figure 4) (*p* < 0.05).

### 3.5. Effect of CTL and Kaempferol on Gluconeogenesis and Hepatic Insulin Resistance in the ER Stress-Induced HepG2 Cells

**Figure 5:**
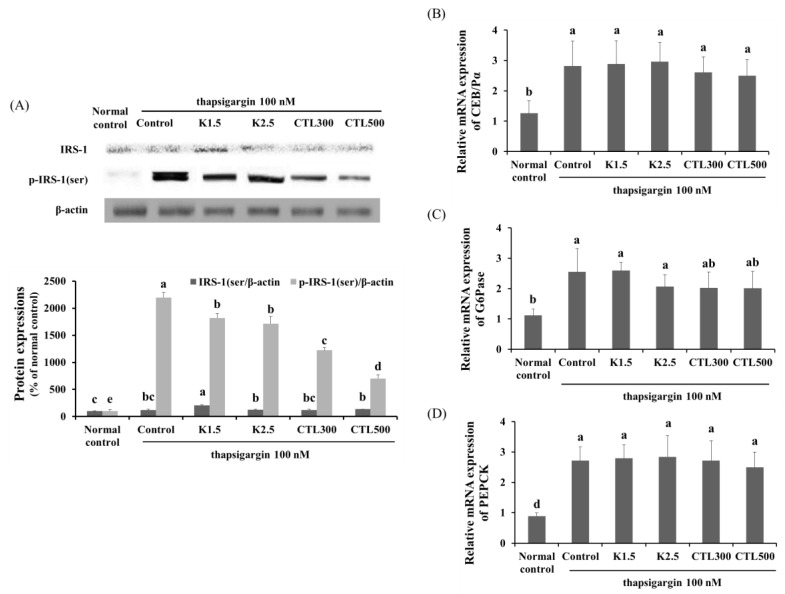
Effect of CTL and kaempferol on gluconeogenesis and insulin resistance in thapsigargin-induced HepG2 cells. (**A**) Representative Western blots for total protein and serine phosphorylation of IRS-1 in thapsigargin-induced HepG2 cells with CT water extract or kaempferol; mRNA expression of (**B**) C/EBPα, (**C**) PEPCK and (**D**) G6Pase in thapsigargin-induced HepG2 cells with CT water extract or kaempferol. The data are expressed as the mean ± SD (*n* = 3). The different letters show a significant difference at *p* < 0.05 as determined by Duncan’s multiple-range test.

The expression of phosphorylated IRS-1 serine was determined by Western blot analysis, and mRNA expression of C/EBPα, PEPCK and G6Pase was determined by real-time PCR in thapsigargin-induced HepG2 cells with CT water extract or kaempferol. The expression of IRS-1 serine phosphorylation, C/EBPα, PEPCK and G6Pase increased significantly in the thapsigargin-stimulated control group compared to the normal control group. The expression of IRS-1 serine phosphorylation decreased significantly in the thapsigargin-stimulated HepG2 cells treated with kaempferol and CTL compared to the thapsigargin-stimulated control group. However, the K1.5, K2.5, CTL300 and CTL500 treatments caused no statistically-significant difference in the level of mRNA expression of C/EBPα, PEPCK or G6Pase compared to the thapsigargin-stimulated control group (Figure 5) (*p* < 0.05).

## 4. Discussion

Hepatocytes require a large and well-developed ER, and they are sensitive to alterations in ER homeostasis [23]. Hepatic ER stress is associated with several metabolic conditions, including increased gluconeogenesis and the production of cytokines. Increasing evidence shows that hepatic ER stress is associated with the pathogenesis of several liver diseases and metabolic disorders, including steatosis and hepatic insulin resistance [6,23,24,25]. Hepatic ER stress-induced insulin resistance also plays a central role in the pathogenesis of type 2 diabetes mellitus [25].

In this study, we investigated the effects of CTL on ER stress-induced inflammation and hepatic insulin resistance in HepG2 cells. We also quantitated the level of kaempferol in CTL, and we investigated the effects of kaempferol on ER stress-induced inflammation and hepatic insulin resistance in HepG2 cells. We found that the concentration of kaempferol was 5.07 ± 0.08 mg/g in CTL. We decided that 300 μg/mL and 500 μg/mL of CTL have sufficient effects and no signs of cytotoxicity in the *in vitro* assay. Thus, we investigated the effects of different levels of kaempferol (1.5 μg/mL and 2.5 μg/mL) and CTL (300 μg/mL and 500 μg/mL) on ER stress-induced inflammation and hepatic insulin resistance in HepG2 cells.

During the accumulation of unfolding proteins in the ER, the UPR is initiated by three ER transmembrane proteins (PERK, ATF6 and IRE1α). The PERK/eIF2 UPR pathway reduces the ER workload by preventing the production of newly-synthesized proteins and reducing the accumulation of proteins within the ER lumen [5,26]. ATF6 activation induces its transfer from the ER to the Golgi, which leads to the release of the N-terminal ATF6 fragment (ATF6-N). ATF6-N enters the nucleus and binds to the ER stress-response element (ERSE), which activates the expression of ER chaperone proteins to aid in protein folding and ER-associated degradation. The XBP-1, mediated by IRE1α, binds to ERSE and activates the expression of chaperones [27,28]. However, when these corrective actions of UPR are insufficient to attenuate ER stress and the ER stress response is prolonged, the UPR switches to a cell death mechanism. The PERK/eIF2α/ATF4 UPR pathway and the IRE1α/JNK UPR pathway induce the expression of CHOP, which is a proapoptotic transcription factor [8,9,29].

In the present study, we used thapsigargin to stimulate ER stress in HepG2 cells. Thapsigargin is a noncompetitive inhibitor that can block the ER calcium ATPase pump [30]. We found a marked increase in the activation of the ER stress response in the thapsigargin-stimulated control group due to increases in the phosphorylation of eIF2α, IRE1α and JNK, as well as increases in the mRNA expression of ATF4 and CHOP compared to the normal group. In addition, the mRNA expression of XBP-1 and the ER chaperone protein (GRP78) was significantly increased in the thapsigargin-stimulated control group compared to that in the normal group (*p* < 0.05). Naem *et al.* [31] showed that thapsigargin induces ER stress in HepG2 cells via phosphorylation of eIF2α and JNK and that GRP78 is expressed in a dose-dependent manner. The increased expression of GRP78 can be used as a biological marker for the onset of the UPR [32]. The kaempferol treatment causes significant decreases only in the levels of ER stress proteins and ATF4 expression (*p* < 0.05). In the report by Kim *et al.* [33], they reported that 10 μM of kaempferol inhibit the expression of ER stress proteins (GRP78, ATF-6, XBP-1, eIF2α and IRE1α) and CHOP in ischemia-reperfusion-exposed cardiac cells. Thus, the concentration of kaempferol in our experiment may have been insufficient to suppress the ER stress.

Some studies have suggested that ER stress induces the inflammation response and that chronic inflammation substantially contributes to the development of several diseases, including arthritis, Alzheimer’s disease, diabetes and cardiovascular disease [34,35]. Many studies have demonstrated that the induction of ER stress increases the expression of pro-inflammatory cytokines in cellular systems [24,34,35]. Other studies have shown that ER stress and the UPR are linked to inflammatory signaling networks, such as the JNK and NF-κB pathways [6,10]. The present study revealed a marked increase in the expression of NF-κB phosphorylation and pro-inflammatory cytokines in the ER stress-induced HepG2 cells compared to their expression in normal HepG2 cells. In contrast, the levels of NF-κB phosphorylation and proinflammatory cytokines (except for IL-6) in the ER stress-induced HepG2 cells treated with CTL decreased significantly compared to those in the thapsigargin-stimulated control group. None of the proinflammatory cytokines’ levels (TNF-α, IL-1β and IL-6) changed significantly in the ER stress-induced HepG2 cells treated with kaempferol compared to those in the thapsigargin-stimulated control group (*p* < 0.05).

García-Mediavilla *et al.* [36] demonstrated that flavonoids, such as quercetin and kaempferol, significantly inhibit the mRNA level of CRP and the activation of NF-κB in a human hepatocyte-derived cell line. Jeong *et al.* [37] found that CT suppresses the production of TNF-α and IL-1β and the degradation of I kappa B-alpha in RAW264.7 macrophages. Joo *et al.* [38] showed that glycoproteins of CT suppress the expression of inflammatory-related proteins through regulation of the NF-κB pathway. According to these reports, we suggest that quercetin and glycoproteins in CTL, and not only kaempferol, can suppress inflammation by inhibiting the activation of NF-κB.

**Figure 6:**
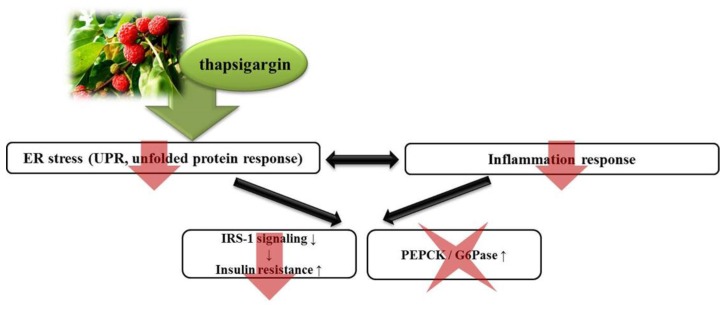
Effect of CTL on ER stress-induced inflammation and hepatic insulin resistance. The present study indicates that CTL can improve hepatic insulin resistance via suppression of ER stress-induced inhibition of IRS signaling and the inflammation response, but that they do not interrupt the C/EBPα/gluconeogenic gene pathway.

Several studies have shown that severe ER stress and ER stress-induced pro-inflammatory cytokines lead to serine phosphorylation of IRS and the development of insulin resistance [39]. ER stress-induced IRE1α phosphorylation and pro-inflammatory cytokines activate the phosphorylation of JNK [40]. The activation of JNK inhibits IRS signaling during insulin stimulation [41,42,43]. In addition, the phosphorylation of eIF2α leads to the activation of C/EBPs, which results in the expression of gluconeogenic genes, such as PEPCK or G6Pase [44,45,46]. Thus, hepatic ER stress plays a role in hepatic insulin resistance and metabolic dysregulation via inhibition of IRS signaling and activation of gluconeogenesis. We found a significant increase in serine phosphorylation of IRS-1, which is a key negative-feedback control mechanism and the expression of C/EBPα and gluconeogenic genes, such as PEPCK and G6Pase, in the thapsigargin-stimulated control group. We also demonstrated that the kaempferol and CTL treatments of the ER stress-induced HepG2 cells resulted in significant decreases in serine phosphorylation of IRS-1 compared to the thapsigargin-stimulated control group (*p* < 0.05). However, there was no statistically-significant difference in the level of mRNA expression of C/EBPα, PEPCK or G6Pase in the ER stress-induced HepG2 cells of the groups treated with kaempferol and CTL compared to that in the thapsigargin-stimulated control group. These observations indicate that kaempferol and CTL interrupt IRE1α/JNK/serine phosphorylation of the IRS-1 pathway, but that they do not interrupt the C/EBPα/gluconeogenic gene pathway (Figure 6).

In the present study, we found that CTL and kaempferol can reduce hepatic insulin resistance via suppression of ER stress-induced inhibition of IRS signaling and the inflammation response, but that they do not interrupt the C/EBPα/gluconeogenic gene pathway. However, whether these affect glucose production and/or uptake remains to be determined.

## 5. Conclusions

In the present study, we quantitated kaempferol in CTL and investigated the effects of kaempferol and CTL on ER stress-induced inflammation and hepatic insulin resistance in HepG2 cells. We used thapsigargin to simulate ER stress in the HepG2 cells. We found a marked increase in the activation of the ER stress response, inflammation, and insulin resistance in the thapsigargin-stimulated control group. The CTL treatment interrupted the ER stress response, inflammation, and serine phosphorylation of IRS-1. Kempferol also partially inhibited the ER stress response, inflammation, and insulin resistance. However CTL and kaempferol led to the suppression of serine phosphorylation and did not interrupt the C/EBPα/gluconeogenic gene pathway. In conclusion, we suggest that CTL, mediated by kaempferol, can protect against ER stress-induced inflammation and insulin resistance in the liver.
